# Pregnancy in the Aged

**Published:** 1873-12

**Authors:** 


Pregnancy in the Aged.—Dr. Meynert has communicated to us the following case which has fallen under his own observation: "A lady died at the age of eighty-five years, having had four accouchements. The first took place at the age of forty; the second, at forty-eight; the third, at fifty-one, and the fourth, at fifty-six. Five girls were born, of whom three are still living, the two twins being seventy-seven years old, and the youngest child seventy-one years. These three persons, the two eldest of whom have been married and have several children, still enjoy the most excellent health."—*Lyon Medicale.*

